# Supplementary figures and images for: Retention in care and predictors of attrition among HIV-infected patients who started antiretroviral therapy in Kinshasa, DRC, before and after the implementation of the ‘treat-all’ strategy
Source: PLOS Glob Public Health. 2022 Mar 11;2(3):e0000259. doi: 10.1371/journal.pgph.0000259 (PMC10022330; doi:10.1371/journal.pgph.0000259)

**S1 Fig**


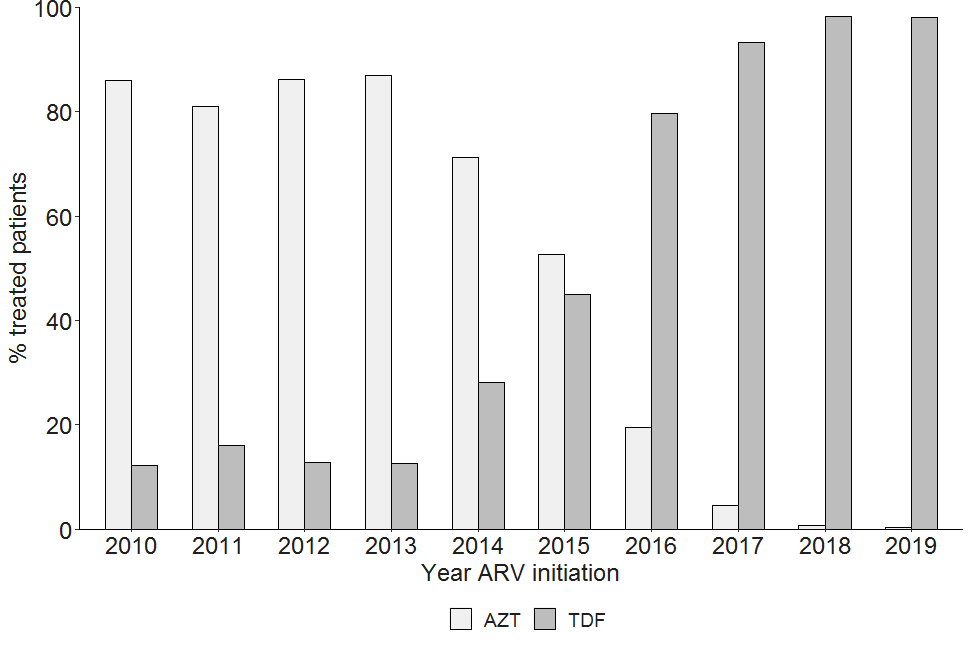

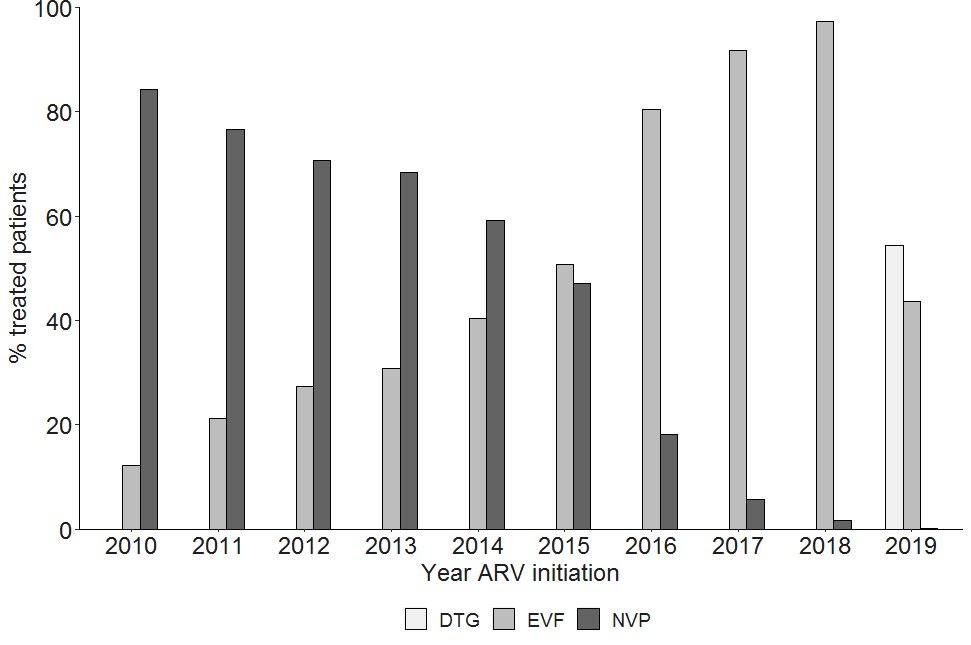


S1 Fig. Evolution of ARV treatment at initiation.

Supplement: S1 Fig — (DOCX) [file pgph.0000259.s001.docx]

**S2 Fig**


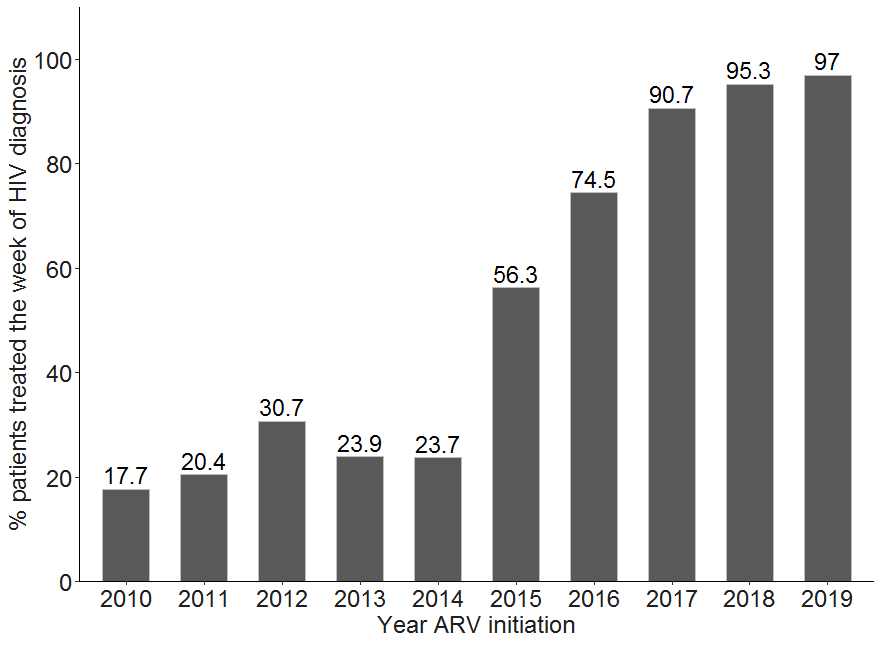


**S2 Fig**: Evolution of the proportion of PLWH who started ART the week of HIV diagnosis

Supplement: S2 Fig — (DOCX) [file pgph.0000259.s002.docx]
