# Supplementary material for: Retention in care and predictors of attrition among HIV-infected patients who started antiretroviral therapy in Kinshasa, DRC, before and after the implementation of the ‘treat-all’ strategy
Source: PLOS Glob Public Health. 2022 Mar 11;2(3):e0000259. doi: 10.1371/journal.pgph.0000259 (PMC10022330; doi:10.1371/journal.pgph.0000259)
Supplement: S1 Table — (DOCX) [file pgph.0000259.s003.docx]

| **S1 Table.** | | | |
| --- | --- | --- | --- |
| Time since ART initiation | Total number of patients at risk | Cumulative number of LTFUs or deaths | Estimated retention (95% CI) |
| 1 month | 15716 | 905 | 0.94 (0.94 ; 0.95) |
| 3 months | 14505 | 1250 | 0.92 (0.92 ; 0.92) |
| 6 months | 14003 | 1772 | 0.89 (0.88 ; 0.89) |
| 1 year | 12819 | 2561 | 0.83 (0.83 ; 0.84) |
| 2 years | 8402 | 3334 | 0.77 (0.77 ; 0.78) |
| 3 years | 4933 | 3811 | 0.71 (0.71 ; 0.72) |
| 4 years | 3148 | 4138 | 0.66 (0.65 ; 0.67) |
| 5 years | 1884 | 4356 | 0.59 (0.58 ; 0.60) |
| 6 years | 1099 | 4516 | 0.53 (0.52 ; 0.55) |
| 7 years | 530 | 4597 | 0.48 (0.46 ; 0.49) |
| 8 years | 209 | 4643 | 0.41 (0.39 ; 0.44) |
| 9 years | 95 | 4657 | 0.37 (0.34 ; 0.40) |
| 10 years | 42 | 4664 | 0.32 (0.28 ; 0.37) |

**S1 Table.** Retention (N=15762 PLWH patients)
