# Supplementary material for: Retention in care and predictors of attrition among HIV-infected patients who started antiretroviral therapy in Kinshasa, DRC, before and after the implementation of the ‘treat-all’ strategy
Source: PLOS Glob Public Health. 2022 Mar 11;2(3):e0000259. doi: 10.1371/journal.pgph.0000259 (PMC10022330; doi:10.1371/journal.pgph.0000259)
Supplement: S2 Table — (DOCX) [file pgph.0000259.s004.docx]

| **S2 Table.** | | | | | | |  |
| --- | --- | --- | --- | --- | --- | --- | --- |
|  | ART initiated <Nov 2016 (N=4481) | | | ART initiated ≥Nov 2016 (N=11281) | | | |
| Time since ART initiation | Total number of patients at risk | Cumulative number of LTFUs or deaths | Estimated retention (95% CI) | Total number of patients at risk | Cumulative number of LTFUs or deaths | Estimated retention (95% CI) | |
| 1 month | 4264 | 270 | 0.94 (0.93 ; 0.95) | 10772 | 635 | 0.94 (0.94 ; 0.95) | |
| 3 months | 4081 | 387 | 0.91 (0.90 ; 0.92) | 10424 | 863 | 0.92 (0.92 ; 0.93) | |
| 6 months | 3931 | 525 | 0.88 (0.87 ; 0.89) | 10072 | 1247 | 0.89 (0.88 ; 0.90) | |
| 1 year | 3716 | 697 | 0.84 (0.83 ; 0.85) | 9103 | 1864 | 0.83 (0.82 ; 0.84) | |
| 2 years | 3442 | 904 | 0.80 (0.78 ; 0.81) | 4960 | 2430 | 0.76 (0.75 ; 0.77) | |
| 3 years | 3205 | 1131 | 0.74 (0.73 ; 0.76) | 1728 | 2680 | 0.69 (0.68 ; 0.70) | |
| 4 years | 2868 | 1422 | 0.67 (0.66 ; 0.69) | 605 | 2716 | 0.67 (0.66 ; 0.69) | |
| 5 years | 1883 | 1639 | 0.61 (0.60 ; 0.63) | - | - | - | |
| 6 years | 1099 | 1799 | 0.54 (0.53 ; 0.56) | - | - | - | |
| 7 years | 530 | 1880 | 0.49 (0.47 ; 0.51) | - | - | - | |
| 8 years | 209 | 1926 | 0.42 (0.40 ; 0.45) | - | - | - | |
| 9 years | 95 | 1940 | 0.38 (0.35 ; 0.41) | - | - | - | |
| 10 years | 42 | 1947 | 0.33 (0.29 ; 0.38) | - | - | - | |

**S2 Table.** Retention before and after November 2016 (N=15762)
