# Supplementary material for: Retention in care and predictors of attrition among HIV-infected patients who started antiretroviral therapy in Kinshasa, DRC, before and after the implementation of the ‘treat-all’ strategy
Source: PLOS Glob Public Health. 2022 Mar 11;2(3):e0000259. doi: 10.1371/journal.pgph.0000259 (PMC10022330; doi:10.1371/journal.pgph.0000259)
Supplement: S3 Table — (DOCX) [file pgph.0000259.s005.docx]

| **S3 Table** | | | | | | |  |
| --- | --- | --- | --- | --- | --- | --- | --- |
|  | ART initiated <Nov 2016 (N=4481) | | | ART initiated ≥Nov 2016 (N=11281) | | |  |
|  | N | HR (95% CI) | p value | N | HR (95% CI) | p value |  |
| Sex, female | 4468 | 0.99 (0.89 ; 1.1) | 0.82 | 11248 | 0.95 (0.87 ; 1.03) | 0.24 |  |
| Pregnant women | 2971 | 1.0 (0.85; 1.2) | 0.84 | 7380 | 1.2 (0.97; 1.4) | 0.10 |  |
| Age <25 years (ref ≥25) | 4468 | 1.3 (1.1; 1.5) | 0.0035 | 11248 | 1.1 (0.97; 1.3) | 0.15 |  |
| Weight (kg) | 3689 | 0.97 (0.97; 0.98) | <0.0001 | 9897 | 0.98 (0.97; 0.98) | <0.0001 |  |
|  |  |  |  |  |  |  |  |
| CD4 >350 cells/mm³ | 2257 | 1.1 (0.93 ; 1.3) | 0.30 | 175 | - | - |  |
| WHO stage III/IV (ref=I/II) | 3364 | 1.2 (1.1; 1.3) | 0.0012 | 8633 | 1.4 (1.3; 1.5) | <0.0001 |  |
| Advanced HIV^(a)^ | 3762 | 1.1 (0.98; 1.3) | 0.11 | 8690 | 1.2 (1.1; 1.3) | 0.0025 |  |
|  |  |  |  |  |  |  |  |
| Initial ARV treatment |  |  |  |  |  |  |  |
| NRTI : TDF (=ref) vs. AZT | 4409 | 0.94 (0.86; 1.03) | 0.17 | 11081 | 0.41 (0.27; 0.62) | <0.0001 |  |
| Third drug: NVP (ref=EFV) | 4401 | 0.90 (0.82; 0.98) | 0.020 | 8482 | 0.37 (0.25; 0.54) | <0.0001 |  |
| Third drug: (ref=DTG) | - |  |  | 11060 |  |  |  |
| EFV |  | - | - |  | 1.2 (1.1; 1.3) | 0.0026 |  |
| NVP |  | - | - |  | 0.44 (0.30; 0.65) | <0.0001 |  |
| Days since HIV diagnosis  > 7 days (ref=≤ 7 days) | 3011 | 1.1 (0.94; 1.2) | 0.35 | 8683 | 1.6 (1.4; 1.9) | <0.0001 |  |
|  |  |  |  |  |  |  |  |
| IPT | 3628 | 0.37 (0.20; 0.68) | 0.0016 | 10921 | 1.0 (0.92; 1.1) | 0.66 |  |
| CPT | 4335 | 0.65 (0.58; 0.73) | <0.0001 | 11211 | 0.57 (0.51; 0.64) | <0.0001 |  |
|  |  |  |  |  |  |  |  |
| Last ARV treatment |  |  |  |  |  |  |  |
| NRTI : TDF (=ref) vs. AZT | 4307 | 7.9 (7.1; 8.7) | <0.0001 | 10735 | 4.7 (3.0; 7.3) | <0.0001 |  |
| Third drug: (ref=DTG) | 4297 |  |  | 10728 |  |  |  |
| EFV |  | 14 (13; 17) | <0.0001 |  | 11 (10; 12) | <0.0001 |  |
| NVP |  | 31 (27; 36) | <0.0001 |  | 11 (6.9; 18) | <0.0001 |  |
|  |  |  |  |  |  |  |  |
| Care Center (ref=small) | 4468 |  |  | 11248 |  |  |  |
| Medium |  | 2.2 (1.7; 2.9) | <0.0001 |  | 1.2 (1.03; 1.3) | 0.016 |  |
| Large |  | 3.9 (3.0; 5.1) | <0.0001 |  | 2.0 (1.7; 2.2) | <0.0001 |  |
| *^(a)^ Advanced HIV if CD4<200 cells/mm³ and/or WHO stage III/IV*  *Mixed effects Cox regression model: p value, HR (95% CI).*  *IPT : isoniazid prophylaxis treatment; CPT: cotrimoxazole prophylaxis treatment* | | | | | | |  |

**S3 Table**. Characteristics associated with retention: Mixed effects Univariate Cox regression models on probability of death or LTFU for the entire cohort for patients treated before or ≥ Nov 2016
