# Supplementary material for: Retention in care and predictors of attrition among HIV-infected patients who started antiretroviral therapy in Kinshasa, DRC, before and after the implementation of the ‘treat-all’ strategy
Source: PLOS Glob Public Health. 2022 Mar 11;2(3):e0000259. doi: 10.1371/journal.pgph.0000259 (PMC10022330; doi:10.1371/journal.pgph.0000259)
Supplement: Attachment — Submitted filename: Reply to reviewers FINAL.docx [file pgph.0000259.s006.docx]

**Reviewer #1**: Review of PGPH-D-21-00142

Summary: Using health records from thousands of patients in Kinshasa, the effect of early vs later ART initiation was explored on retention/attrition of patients.

Major comments:
-I think the premise that the Treat All guidelines may increase healthcare work and perhaps impact ability of clinics to follow-up on patients. Data are mixed and so this is the reason for this study. Perhaps this could be outlined more clearly and succinctly in the introduction.

We agree with the reviewer. We modified the introduction to emphasize this point in the revised manuscript.

This seems like a valid study to help with this question but there are a number of limitations.
-Is all patient care documented in Tier.Net? is this used across the DRC? Does this also record deaths? If people went to different facilities would it be captured? I think more details on your main data source with be helpful.

We thank the reviewer for this comment. It was made by reviewer 2 and the editors as well. The manuscript has been largely modified to include more details regarding data collection and methodology.

TIER.Net is an electronic patient management system with modules to capture patient-level data on HIV counselling and testing, pre-ART and ART services. The use of this tool is indeed strongly recommended in DRC and in other countries in Africa. The implementation of TIER.Net across DRC is ongoing. The use of Tier.Net is increasing but is not completely widespread yet, including in Kinshasa.

In principle, deaths are included in Tier.Net. Nevertheless, the update process is sometimes delayed and it should be stressed that not all events are captured. LTFU patients are not traced and it is therefore possible that a proportion of LTFU is due to unrecorded deaths.

Transfers to other facilities may be missed if this information is not provided by the patients. There is no unique identification code shared by all facilities, and not all facilities use the electronic data base as well. For those reasons, transfers to others facilities could have been underestimated in our study.

-More details on the clinics that these data are taken from would also be helpful—are these the main sources of HIV care in Kinshasa? Where else do people get HIV care from?

The 85 facilities included in the analysis are not the main sources of HIV care in Kinshasa. They represent six health care zones out of 18 that are supported by PEPFAR and ICAP. The Global Fund supports other health care zones (17), for a total of 35 healthcare zones. Details have been included in the revised manuscript.

Are there guidelines about following up clients who miss appointments? Is this standard already?

We thank the reviewers for this comment. Several strategies are implemented to reduce attrition: support from relay person in the community, peer advisers, adherence clubs. The implementation of such approaches is recommended of the national plan against HIV/AIDS. Nevertheless, they are not used in all facilities and there are no common standardized strategies to reduce LTFU so far. This has been stated in the revised manuscript.

-Did guidelines change for INH and cotrimoxazole provision? Is there another reason for the large difference between P1 and P2?

Indeed, WHO guidelines have been modified. Co-trimoxazole prophylaxis was initially recommended for adults (including pregnant women) with severe or advanced HIV clinical disease (WHO stage 3 or 4) and/or with a CD4 count of ≤350 cells/mm3. In settings where malaria and/or severe bacterial infections are highly prevalent, co-trimoxazole prophylaxis should now be initiated regardless of CD4 cell count or WHO stage. Regarding TB, guidelines also evolved and recommend that adults and adolescents living with HIV who are unlikely to have active TB should receive TB preventive treatment as part of a comprehensive package of HIV care.

This has been included in the manuscript for clarity.

-Were you able to determine if attrition was LTFU versus death? Are deaths recorded in a central location?

In principle, deaths should be included in Tier.Net. Nevertheless, the update process is sometimes delayed and it should be stressed that not all events are captured. LTFU patients are not traced and it is therefore possible that a proportion of LTFU is due to unrecorded deaths (see above).

-I am finding it confusing to understand that some retention was improved but that overall attrition was worse in P2 compared to P1…

We thank the reviewer for this important comment. Methodology and results presentation were adapted to be less confusing with this comparison and to take into account the proportional hazard assumption of the Cox models. Overall retention from 0 to 4 years after treatment initiation is not statistically different between P1 and P2 cohorts (Cox regression model *: p= 0.39). Figure 4 and table 2 have been adapted.

We believe that this new way to present the data is clearer and we hope that it will satisfy the reviewer.

* Since the proportional hazard assumption is not met, model was limited to 0 to 48 months after treatment initiation, and adjusted with a “time x cohort” term.

-There are some grammatical errors and missing words throughout that would benefit from perhaps a third-party editor.

The manuscript has been edited by The SNAS (Springer Nature Author Services) Support Team, as requested. The certificate has been transmitted to the editors.

-why would you think that INH prophylaxis or clotrimaxole would be associated with LTFU?

This is a very good point raised by the reviewer. We performed additional analysis in order to analyze whether the impact on retention might be due to death and/or to LTFU.

| **Table. Effect of INH prophylaxis or cotrimoxazole on probability of death or of LTFU (Simple Cox regression models)** | | | | | |
| --- | --- | --- | --- | --- | --- |
|  |  | N | HR (95%CI) | p-value |  |
| Death or LTFU | IPT | 14595 | 0.94 (0.85 ; 1.03) | 0.19 |  |
| Death | IPT | 14595 | 0.41 (0.29 ; 0.58) | <0.0001 |  |
| LTFU | IPT | 14595 | 1.0 (0.94 ; 1.2) | 0.39 |  |
|  |  |  |  |  |  |
| Death or LTFU | CPT | 15592 | 0.66 (0.61 ; 0.71) | <0.0001 |  |
| Death | CPT | 15592 | 0.70 (0.58 ; 0.85) | 0.0002 |  |
| LTFU | CPT | 15592 | 0.65 (0.60 ; 0.71) | <0.0001 |  |
| *IPT: isoniazid prophylaxis treatment*  *CPT: cotrimoxazole prophylaxis treatment* | | | | | |

The impact of INH is only due to a reduction of death which can likely be explained by a reduction of infections including TB. The impact of CPT is clear for both Death and LTFU. Although the effect on death may reflect a reduction of death due to infections such as pneumocystis or malaria, the effect on LTFU is indeed unclear. One can speculate that providing to the patients a global HIV care package and not ART only may increase adherence and trust to the healthcare system. Another possibility is that patients accepting to take prophylaxis are from the beginning the patient who trust the healthcare system and who are willing to have a sustained follow up. This has been included in the discussion.

-there are obviously way more patients in P1 compared to P2—do you think this is a limitation? Should you have tried to match the number of patients in P1 compared to P2? Similar follow-up time is very different for the populations and could this have impacted your results?

We thank the reviewer for this interesting comment. We do not believe that this is major limitation of our study. Indeed, the sample size is large in both P1 and P2 groups (Total: N=15762, P1: 4481, P2:11281 PLWH patients). It is true that P1 includes fewer patients compared to P2 and this retrospective study design led to follow-up time higher in P1 compared to P2.

Nevertheless, the 95% confidence intervals of the estimated retention (Table 3) are narrow in the two cohorts. We thus consider that the lower sample size in P1 do not affect the comparison in this table. Follow-up time in the P2 cohort does not allow for comparison beyond 4 years after initiation of treatment. For that reason, the analysis of the impact of timing of ART initiation (P1 or P2) in Figure 4 and in table 2 have been adapted and limited to the first 4 years after treatment initiation.

The 95% confidence intervals on the estimated Hazard Ratio (Table S2) are narrow in the two cohorts and we also consider that the lower sample size in P1 do not affect the comparison in this table.

-were any of your analyses adjusted? I could imagine that poor baseline CD4 or functional status could be associated with death or ‘attrition’.

Analyses were not adjusted, we used simple (=univariate) Cox regression models. We did not consider multivariate analysis due to the presence of a large number of missing data for some variables of interest that would have reduced the number of observations too strongly. For instance, CD4 cell count at baseline was not performed in recent years (see our recent publication PMID: 33369905).

Statistical analysis methodology section has been adapted to be clearer about this point.

-In results, you refer to figures and tables. I would also write out the results you are trying to highlight from your figures and tables for easy of reading and to really highlight the main results of the paper.

The results section has been modified accordingly.

-does it make sense to define attrition as both death and loss to follow-up? These seem like very different measures and have different mechanisms—perhaps the healthier patients are lost to follow-up as they move?

We do agree with the reviewer and we feel this is an important point. We thus provided additional data regarding LTFU only in the revised manuscript. Regarding LTFU analysis, Table 3 has been added in the revised manuscript as well as a section in the results. Interestingly, factors associated with attrition are similar to factors associated with LTFU.

-in figure 4, which line is associated with better retention? The lines cross and then appear to swop positions.

This is a good point raised by the reviewer.

Methodology and results presentation were adapted to be less confusing with this comparison.

Overall retention from 0 to 4 years after treatment initiation is not statistically different between P1 and P2 cohorts (Cox regression model *: p= 0.39). Figure 4 and Table 2 has been adapted.

** Since the proportional hazard assumption is not met, model was limited to 0 to 48 months after treatment initiation, and adjusted with a “time x cohort” term.*

-I think because the sample size is large, you have power to see even very small differences in retention but I think the question is are these clinically relevant and meaningful given such small effect sizes?
Indeed, the sample size is large. This is a great strength of our study. It is true that larger sample size increase power to see smaller differences in retention (leading to smaller p-values). In other words, it allows identifying factors associated with attrition, some of them having a smaller influence then other.

Regarding relevance of factors associated with retention in care, hazard Ratios are also presented in the Tables, those measure the impact of each variable on retention and are more informative than p-values.

Minor comments;
-Please make sure to define abbreviations prior to use: PLWH, DRC.

Abbreviations have been added. Thank you.
-Intro: Is the 68.4% of PLWH on ART in June 2020 a global statistic?

Yes it is. Reference is indicated in the manuscript.

<https://www.unaids.org/fr/resources/fact-sheet>
-First sentence of second intro paragraph is hard to follow. I think you need to first lay out that there might be work overload with increased ART.

The sentence has been edited.
-in methods, what is the difference between the Tier.Net collected data and ‘programmatic data’

Programmatic data include population data extracted from individual data.
-last sentence in discussion is not a complete sentence.

The sentence has been edited.
-Probably wouldn’t include coefficient+/- SE in the tables—HRs and 95% CI are more standard.

Coeffients +/- SE were removed from the tables.

**Reviewer #2**: The authors describe ART care outcomes in DRC. It provides programmatic estimates of retention as well as assessment of factors associated with attrition. Findings of this operational research study are important for the context of DRC and this part of Africa and worth publishing.
This article however has several limitations that need improvements. I tried to give some advice below how it could be improved.


Major considerations
While the text is overall written well, it would benefit from language editing and several sections could be more concise.

The manuscript has been edited by The SNAS (Springer Nature Author Services) Support Team, as requested. The certificate has been transmitted to the editors.

Abstract: I think better to expand the Result section and shorten the Conclusions. There are a lot of interesting results in the main text that could be presented under results of the abstract.

The abstract has been modified as requested.

1 . Introduction:
Define more clearly what treat-all means, such as ART initiation at the time of diagnosis irrespective of immunological criteria.

The reviewer is right. TA strategy is based on the recommendation that antiretroviral medicines should be prescribed to people as soon as possible after their HIV diagnosis regardless of their CD4 count. We have added this sentence in the introduction.

2. Methods/ study design:
It would be good to have a bit more information about the facilities included in the study. For instance, private vs public sector; supported by other organizations (NGOs, PEPFAR)?

This was requested by reviewers 1 as well (see above). We have included this information in the revised manuscript.

The cohorts of P1 and P2 need better definition. The author mentions that it depended on the timing of ART initiation. I assume this statement refers to treatment eligibility criteria. It is important to know what these treatment eligibility criteria were; for instance CD4<350 vs treat-all (ART initiation irrespective of the CD4 cell count threshold”

We thank the reviewer for this comment. We already better defined what is the “treat all” strategy in the introduction, as requested. We also indicated what were the eligibility criteria before the TA strategy, from 2010 to 2016, in the method section.

3/ Methods/ analysis:
This section should be clearer what happened to patients who were transferred out. It is mentioned later that they have been removed from analysis, but this should already be stated in this section. Related to TFO, I do not understand why TFO were removed from analysis. Cox regression and Kaplan Meier analysis allows for censoring of TFO and therefore with the benefit of not reducing the sample size.

We agree with the reviewer. We thus decided to keep transferred-out patients to the study cohort (see modified figure 1 in the revised manuscript). All the values thus changed in the revised figures and tables. However, the impact was not significant on the results/conclusions as the number of TFO is low in comparison to sample size.

Throughout the article, it is not clear to me if you calculate crude/unadjusted HRs or present adjusted HR (with all variables included into one model). This needs to be more clear as its interpretation would make a difference.

This point was made by reviewer 1 as well (see above). Analyses were not adjusted, we used simple (=univariate) Cox regression models. We did not consider multivariate analysis due to the presence of a large number of missing data for some variables of interest that would have reduced the number of observations too strongly (including baseline CD4). Statistical analysis methodology section has been adapted to be clearer about this point.

This part should also describe that one model was build for the entire cohort, and then separate models were build for P1 and P2 (as according to Table 2 and TableS2.

Statistical analysis methodology section has been adapted to be clearer about this point.

The KM graphs (Figure 3, Advanced HIV disease) suggests that the proportional hazard assumption may not be met. The assumption of the Cox model is that the hazards are proportional (PH), and violation of the PH assumption may lead to biased effect estimates in Cox regression analysis. Did you test for this assumption? If there is a violation, it should be addressed in analysis or should at least be mentioned under limitations.

Proportional hazard assumption was tested for all the variables. It was not met for advanced disease (Figure 3B) and for P1 versus P2 (Figure 4). To address this assumption violation, Cox models for the two affected variables have been adjusted with a “time x subgroup” variable in Table 2 and the model for P1 versus P2 comparison was limited to 0 to 48 months after treatment initiation.

To avoid misinterpretation, all the p-values have been removed from Figures 3 and 4.

Overall, the section should be a bit more clear what was really done: Assessment of crude programmatic outcomes (e.g. retention) of the entire cohort and separately for P1 and P2. And evaluation of predictors of all cause attrition (LTFU and death combined) for the entire cohort and separately for P1 and P2. In case you do not want to present all results of these analyses in the main text, it should be more clear what is the main analysis and what is supplementary analysis.

We agree with the reviewer. We reordered the figures and tables to make the manuscript clearer and easier to read and to follow.

4/ Results:
You report on coef. ± SE and on HR (95% CI). Probably fine not to report on coef. ± SE as the HR is already reported on.

Coeffients +/- SE were removed from the tables.

Table 2. What does “Simple cox regression” mean ? Is it univariate or multivariate analysis?

“Simple Cox regression models” is the same as “univariate”. Statistical analysis methodology section has been adapted to be clearer about this point.

Tables and text: Good to be consistent with reporting of estimates: for instance you report estimates with one, two or three decimal points (see HR) or sometimes with no decimal point. I think better to report the HR with 2 decimal points, proportions with one and to be consistant. By reporting with three decimal points, I think you want to show that some variables are significantly associated with the outcome. That is not really necessary as you already report on the p values.

We do not agree with the reviewer. There is not willingness to present the data in such a way that would favor some outcomes. We would like to stress that we are extremely cautious and rigorous with the statistical analysis.

All results were expressed with 2 significant digits, as it is often requested (ex.: 0.23 or 0.023 or 2.3, …). 95%CI on Hazard Ratios were reported with more than 2 significant digits when necessary to avoid rounding doubts when we are around the value of 1 (ex.: 1.1 (1.03 ; 1.2) because 1.0 can be the rounded value of 0.999).

Figure 1: Good not to report only the numbers but also the percentages. In addition, the last 2 boxes mention patients deceased and patients LTFU. Add one box for patients retained in care.

One box for patients retained in care has been added.

We did not add global percentage of death and LTFU, which can lead to misinterpretation.

Table S1 shows time-evolution of the estimated retention percentages.

Figure 2: re-phrase the title of this Figure. Also mention that it is for the entire cohort.

We modified the manuscript as requested.

Figure 3 has 2 titles: Factors associated with attrition vs Retention evolution …

This has been modified.

For the Figures with the KM plots, maybe to rename them to something like: Kaplan Meir plots of retention since ART initiation for the entire cohort / for P1 and P2

This has been modified.

Table S2: It is not clear why CD4 cell count and WHO staging is not presented in the model/table (see right part of the table for P2).

CD4 cell count were missing for too much observations during P2. This is a worrying observation that was recently published by others and by us (PMID: 33369905).

Model on WHO staging during P2 was added, as requested.

I think in your analysis it would be more correct to categorize CD4 cell count. For instance, patients with very low CD4 (e.g. deaths) and patients with high CD4 cell count (poor adherence) may be more likely lost to care/ death than patients with a CD4 at around 350. This information may get lost if CD4 is modelled as continues variable, but is important for programme manager to see. The introduction also mentions that patients with high CD4 cell count may be less adherent, so got to have a separate category for them.

This is a good point raised by the reviewer.

Log-transformed CD4 values were replaced by the categorical variable CD4 ≤ or >350 in the Cox regression models.

Minor comments:
The abbreviation SD for same day ART is only used once, and is the same as used for standard deviations.
We deleted the abbreviation SD.

This sentence is quite long and could be shortened: “Socio-demographic and clinical characteristics at ART initiation such as sex, age, weight, height, WHO classification, pregnancy, baseline CD4 T cell count, start date of ART, baseline and last ART regimen, Cotrimoxazol and Isoniazid (INH) prophylaxis,” Provide maybe less examples of variables included in analysis as they can also be seen in the tables.

This has been modified.

I think the following sentence needs some modification “The date of LTFU was defined as the most recent visit or the day after the start of ART if the patient presented only at the initiation visit.” to “ … or the day of start of ART ….” In case time zero was the day after start of ART, then maybe indicate that it was “…. one day after the start ….”

This has been modified.

The Discussion starts with a general discussion about retention and TA. I think it would be better to open the Discussion section with a general statement about what you did in the study and what the main findings were.

We shortened the first part of the discussion as requested.

Probably better to refer to “CD4 cell count” than “CD4 T cell count”. The latter abbreviation is not commonly used in programmatic papers.

This has been modified.

Good to be more consistent in the terms used throughout the paper. For instance: ART vs ARV initiated; treat-all vs Treat All vs Treat-All TA vs Test and Treat.

This has been corrected.

You need a reference for this sentence: “Patients followed in big centers were more prone to attrition as previously shown by other.”

This has been added.
