# Supplementary material for: Retention in care and predictors of attrition among HIV-infected patients who started antiretroviral therapy in Kinshasa, DRC, before and after the implementation of the ‘treat-all’ strategy
Source: PLOS Glob Public Health. 2022 Mar 11;2(3):e0000259. doi: 10.1371/journal.pgph.0000259 (PMC10022330; doi:10.1371/journal.pgph.0000259)
Supplement: Attachment — Submitted filename: Reply to reviewers FINAL.docx [file pgph.0000259.s007.docx]

**EDITOR:**

Thank you for submitting your revision. While I can see that the manuscript has improved, I feel there are still some important issues that need addressing before it meets our publication criteria. As there are limited data available on this important issue from your study setting, it would be great to receive an improved version.

Please note that your tracked changes document was not complete (ie not all changes were marked), which has made the review much more time-consuming. For future submissions I would strongly suggest you ensure a complete tracked changes document.

Many of the reviewers’ points were mainly addressed in the rebuttal letter but were hardly implemented in the manuscript. Please could you address these appropriately. These include:

- Details about the facilities included in the analysis

Details have been included in the revised manuscript.

- Details about tier.net (roll-out, usage, recording of deaths etc)

Modifications that were already done during the previous revision are now highlighted in yellow in the present version. Moreover, we have included additional details on Tier.Net implementation in DRC (in tracked changes).

- Rationale for not doing multivariable analysis

As previously explained, we did not initially consider multivariate analysis due to the presence of a large number of missing data for some variables of interest that would have reduced the number of observations strongly.

Nevertheless, we finally decided to follow the suggestions of the editor and the reviewer as we agree that it would strengthen our conclusions to add the multivariate models.

We were surprised to see that although some conclusions remained the same, the impact of the TA implementation appeared to be negative on retention and LTFU.

We thus modified the manuscript according to this result.

- Rationale for limitation to 48 months follow-up in P1 vs P2 analysis

Follow-up time in the P2 cohort does not allow for comparison beyond 4 years after initiation of treatment. For that reason, the analysis of the impact of timing of ART initiation (P1 or P2) have been adapted and limited to the first 4 years after treatment initiation. The statistical analysis section has been updated for clarity.

- Study objectives (last comment of reviewer 3, methods section)

We have augmented this section at the end of the introduction for clarity. We believe that this part of the manuscript fits well to elaborate on the objectives of the study.

- First paragraph of discussion still does not contain the summary of the study’s findings, as is common practice in scientific writing

We included this paragraph in the revised manuscript.

- The information in the methods about the study population (its generalizability), data collection is still insufficient. A lot of information was given in the responses, but much of this was not included in the manuscript or only to a limited degree which does not give sufficient insight into risks of selection and misclassification bias.

Information regarding the included facilities has been included in the methods. Nevertheless, we added in the limits that our observations may not be true everywhere, for instance in less urban area.

**Additional Editor Comments (Editor's review comments):**

Major comments

Abstract:

The abstract does not include the study objective(s).

Objectives have been included.

You phrase your findings in terms of attrition, whereas you present your results in terms of retention. I presume these are the inverse of each other, but this does make it confusing to the reader. I would suggest to stick to the same terminology. This also goes for the rest of the manuscript.

We do agree and we have done the changes where we feel it was unclear.

You include conclusions about findings you did not present in the results (about adolescents and young adults), please make sure to include

Conclusions were changed based on the multivariate analysis.

Introduction: please include your study objectives in the last sentences of your last paragraph. They are currently not explicitly stated anywhere. These should cover all of your analyses, and ideally all results will be presented in the order of the objectives.

We do agree. Changes have been made accordingly.

Methods:

I would suggest to split your study design paragraph into more, it now encompasses much more than just study design (study setting, study population, data collection/data sources, data management/definitions, etc)

Methods section has been divided as requested.

Please elaborate on the generalizability of the selected HIV care facilities in comparison with the other facilities in Kinshasa. (see also my comment above) It might help to include a study setting paragraph to be able to explain this fully. Please also include the role of ICAP (and PEPFAR) in the treatment programmes – how did the treatment and follow-up differ compared to other HIV care centres? This is important to be able to assess the generalizability of your results.

We have created a “study setting” section in the Methods where we added all requested information regarding the generalizability of the results.

You state that Tier.net implementation is ongoing; could you give an indication of how complete this is? Did your site selection have anything to do with this roll-out? And did all the selected facilities have complete data entry for all years and for all their patients, or was only part of their population and/or follow-up data entered?

We have added a couple of sentences for clarity. Tier.net is already widely used in Kinshasa, thanks to the work of data managers in charge of data collection and data encoding. Mobile data managers play a crucial role to collect paper-based records and to encode in Tier.Net. This is not the case yet elsewhere. The implementation of Tier.Net is ongoing in other provinces of DRC.

Participating centers were thus not selected based on Tier.net roll-out, but only on the willingness of centers to collaborate with us.

Why did you not utilize interrupted time series methodology if your main interest is in comparing P1 to P2?

Thank you for this comment. Indeed, interrupted time series methodology can be an interesting method to compare the situation before (P1) and after (P2) the implementation of the ‘treat-all’ strategy. This methodology is well adapted to continuous or binary variables modeling. For example, it is well appropriate to compare P1 to P2 for variables such as time between diagnosis and ARV treatment initiation, or as CD4 counts at ARV initiation, … .

But the outcome studied in this article is the retention, for which survival methods are adapted. In the Cox regression models, a binary ‘cut-off’ variable P1 vs P2 was used.

Why did you not utilize multivariable regression analysis? Please include the motivation for this in your methods section. As this likely introduces major bias in your results, this would need to be featured strongly in the limitation section and in the interpretation of your results.

We have added the multivariate analysis. This resulted in important modifications in the manuscript. The impact of TA implementation appears to be negative on retention in HIV care.

Did you adjust for clustering by study site? This would need to be done in all regression analyses.

We thank the reviewer for the comment. Mixed effects Cox regression models were used to examine each prognostic factor for risk of loss to follow-up or death considering the random care center effect in the revised manuscript.

I struggle with the combining death and LTFU as attrition and looking at the risk factors thereof; I would offer that patients who die are no longer at risk of being LTFU, and the factors associated with dying could be different from those associated with LTFU. Unless you have evidence that the majority of LTFU is in actual fact death? Why not look at risk factors for death and LTFU separately?

We agree with the reviewer. Combining death and LTFU is frequently done in the literature, more specifically in settings where it is difficult to trace LTFU and when deaths are not properly recorded. In principle, deaths are recorded in Tier.Net (this has been discussed in the manuscript). This is the reason why we have already addressed this comment during the previous revision, although we recognize that tracked changes document was unfortunately not complete (our mistake).

We put in green the paragraph in the results part where we investigate factors associated with LTFU after excluding death. We wish to emphasize that the revised “TABLE 3”, in the main manuscript, refers to LTFU only.

What is the hypothesis behind investigating an association between use of CPT or IPT and retention?

We have several hypotheses to answer this question. Indeed, the impact of CPT is clear for both Death and LTFU (see table 2 and table 3). Although the effect on death may reflect a reduction of death due to infections such as pneumocystis or malaria, the effect on LTFU is still unclear.

One can speculate that providing to the patients a global HIV care package and not ART only may increase adherence and trust to the healthcare system. Another possibility is that patients accepting to take prophylaxis are from the beginning the patient who trust the healthcare system and who are willing to have a sustained follow up. This had been included in the discussion during the previous revision. Although we can only make speculations, the results are statistically relevant and we would like to keep it as more explanations could arise later.

Results:

If your main study objective is to compare P1 to P2 (as the introduction suggests), I would suggest you present your findings stratified by both throughout, and move Figure S2 into the main manuscript. It would then also make sense to present all figures/outcomes curtailing your maximum follow-up period, as defined in your methods.

The figure comparing retention in P1 and P2 has been moved to the main manuscript.

Considering the lack of multivariable analysis, I feel that the results of your univariable analysis are overstated. A lot of these differences could be due to confounding by one or more of the other factors.

Multivariate analysis has been included.

I cannot find the analysis leading to the “main” P-value of 0.39 in Table S2, which is what the manuscript refers to.

This p-value is not relevant anymore, due to the modifications of the statistical analysis (moreover, we have to acknowledge that this p-value was wrong in the previous manuscript (should have been 0.039 and not 0.39; please accept our sincerest apologies for this mistake)).

Minor comments

CTX and IPT guidelines: I appreciate that the WHO changed their guidelines, but what was the local guidance on this over time? And what about take up and implementation thereof in practice?

Local guidelines (PNLS for *Plan National de Lutte contre le SIDA*) were adapted to the WHO guidelines. Our data show that the guidelines for CTX and IPT were implemented together with the TA strategy.

**REVIEWER**

Abstract: Please include how you defined retention, as the inverse of attrition or of LTFU? Please include the method of comparing pre/post.

This has been included.

Methods:

- The definition of LTFU is presented in parentheses, please include the source.

Reference has been included

- The term “simple” regression is not standard terminology and can cause confusion (as with reviewer 1), please rephrase to univariable or univariate regression.

This has been changed

Results:

- How come you have so many missing CD4 count values, in particular in P2? Did this have anything to do with the data collection and/or change in guidelines? Please make sure to include this information in the methods section, and in the limitation section. (added later after review of reviewer’s comments: please include the citation to your recent paper on this)

We thank the reviewer for his/her interest. We also believe that the phasing out CD4 cell count was an important finding to publish (PMID: 33369905). We were not the first to do so. Nasuuna and colleagues published similar results from Uganda in *clinical infectious diseases* (PMID: 32373932). CD4 cell count are still in the guidelines (WHO and DRC). We think that the implementation of viral load (VL) testing led to a transfer of resources towards VL, but this is another debate.

As requested, we have included this information in the methods and the limits. Our paper was already cited in the limit section (ref 54).

- The line “in settings where malaria… WHO stage” belongs in methods.

It has been removed from the results.

- Couldn’t the information in Tables S1 and S3 not be presented as Kaplan Meier graphs?

We are not sure to understand this comment as they are presented as suggested in the figures.

- Was the effect of age linear (Fig 3A suggests otherwise)? If not, I would suggest to include age as a categorical variable. If yes, why did you emphasize the risk of attrition particularly in adolescents in the discussion/conclusion?

As suggested, we replaced continuous Age variable by a binary variable (< or ≥ 25 years) in the models.

- The inconsistent use of decimal points is confusing to the reader; I agree with the reviewer that it would be better to stick to two for the HR estimates and one for the proportions. By reporting P values you don’t need to show 0.999 instead of 1.0 in order for the reader to understand.

All results were expressed with 2 significant digits, as it is often requested (ex.: 0.23 or 0.023 or 2.3, …). 95%CI on Hazard Ratios were reported with more than 2 significant digits when necessary to avoid rounding doubts when we are around the value of 1 (ex.: 1.1 (1.03 ; 1.2) because 1.0 can be the rounded value of 0.999).

 Discussion:

- You suggest that taking CPT increases attrition possibly due to patients who accept prophylactic treatment “from the beginning” are more likely to be in follow-up. This suggests that this effect would be stronger in P1 when patients were started on CPT before ART? However, the analysis stratified by P1 and P2 shows no major difference in those estimates. Maybe you could consider editing? (As above, I would suggest many of these effects are due to confounding so I am hesitant to overinterpret them anyway.)

 We understand the point of the reviewer. Nevertheless, the impact of CPT prophylaxis remains statistically relevant in the multivariate analysis. We feel that, although we can only speculate on the reasons for this, it is fair to keep it the analysis.
